# DPP-4 Inhibitors as Treatments for Type 1 Diabetes Mellitus: A Systematic Review and Meta-Analysis

**DOI:** 10.1155/2018/5308582

**Published:** 2018-01-08

**Authors:** Qixian Wang, Min Long, Hua Qu, Rufei Shen, Rui Zhang, Jing Xu, Xin Xiong, Hui Wang, Hongting Zheng

**Affiliations:** Department of Endocrinology, Xinqiao Hospital, Third Military Medical University, Chongqing 400037, China

## Abstract

**Objective:**

Several clinical studies have reported the application of dipeptidyl peptidase-4 (DPP-4) inhibitors as treatments for type 1 diabetes mellitus (T1DM). This study aims to review the outcomes of these existing studies and to discuss the therapeutic effects of DPP-4 inhibitors on T1DM.

**Methods:**

We thoroughly searched the Medline, Embase, PubMed, and Cochrane Library databases and ClinicalTrials.gov for studies concerning the use of DPP-4 inhibitors in patients with T1DM.

**Results:**

In preclinical trials, DPP-4 inhibitors improved the pathogenesis of T1DM. However, only a portion of the studies showed potential efficacy regarding clinical glycemic control and other clinical parameters. From this meta-analysis, pooled data from 5 randomized controlled trials revealed that the additional use of DPP-4 inhibitors resulted in a greater decrease in glycated hemoglobin A1c (HbA1c) levels (0.07%, 95% CI (−0.37%–0.23%)) than insulin monotherapy, although the decrease was not significant. A small decrease in postprandial glucose or insulin consumption was confirmed.

**Conclusion:**

Although DPP-4 inhibitors may be beneficial for T1DM, existing studies do not strongly support these positive effects in clinical practice. Further optimized clinical trials are needed.

## 1. Introduction

Type 1 diabetes mellitus (T1DM) is considered a chronic immune-mediated disease that is characterized by the selective destruction of *β*-cells in genetically susceptible individuals. The proportion of diabetes mellitus patients diagnosed with T1DM is estimated to be 5%–10% [1] with an annual increase of 3.8–5.6% [2–4], but this proportion may be underestimated [5]. Insulin replacement has been the mainstay of therapy for T1DM [6]. New therapies, including immunotherapy, islet transplantation, and stem cell/precursor cell transplantation, have been utilized in attempts to conquer T1DM. However, numerous defects, such as the absence of durable effectiveness, severe adverse effects (e.g., cytokine-release syndrome and transient Epstein-Barr viral infection), immunosuppression, and the scarcity of donors, limit their extended applications in patients with T1DM [7]. Only a small percentage of patients with T1DM achieve the goal of glycemic control. Moreover, treatments that address the underlying disease process are not available. Therefore, explorations of new and efficient therapies for T1DM are urgently needed [8].

Dipeptidyl peptidase-4 (DPP-4) inhibitors, which have been widely used as outstanding blood glucose-dependent antidiabetic agents for patients with type 2 diabetes mellitus (T2DM), show promise. These inhibitors prevent the degradation of incretin (glucagon-like peptide-1 (GLP-1) and glucose-dependent insulinotropic polypeptide (GIP)) by dipeptidyl peptidase-4 enzymes and therefore elevate endogenous GLP-1 levels. GLP-1 stimulates insulin secretion from *β*-cells in a glucose-dependent manner, suppresses glucagon secretion from *α*-cells, and inhibits hepatic glucose production, eventually contributing to the antihyperglycemic effect. In addition, DPP-4 inhibitors preserve the *β*-cell mass [9].

Recently, an increasing number of studies on the topic of DPP-4 inhibitors and T1DM have discovered their mutual characteristics of immune destruction. Insulitis was alleviated in a T1DM animal model treated with DPP-4 inhibitors [10], and the numbers of regulatory T cells, including CD4^+^CD25^+^FoxP3^+^cells, which were reduced in patients with T1DM [11], increased [10]. Glucagon levels have been shown to be decreased during hyperglycemia [12–15] in several clinical trials of T1DM. However, controlled clinical trials have reported controversial effects on postprandial glycemic control, as well as the levels of glycated hemoglobin A1c (HbA1c) and other indicators. Is DPP-4 inhibitor monotherapy or its combination with insulin appropriate for patients with T1DM? Here, we performed a systematic review and meta-analysis to evaluate the roles of DPP-4 inhibitors in T1DM with the aim of directing future clinical trials.

## 2. Methods

### 2.1. Ethical Review

Our research was a study-level systematic review and meta-analysis of clinical trials. Therefore, ethical approval was not necessary for this study.

### 2.2. Search Strategy

This systematic review was performed according to PRISMA guidelines [16]. Two investigators (QX Wang and M Long) thoroughly searched the bibliographic databases Medline, PubMed, Embase, and the Cochrane Library for published articles describing DPP-4 inhibitors and T1DM up to March 10, 2017. In addition, we explored relevant unpublished data at ClinicalTrials.gov. The search strategy used for PubMed (http://www.ncbi.nlm.nih.gov/pubmed/) and the Cochrane Library (http://www.cochranelibrary.com/) was (“dipeptidyl peptidase-4 inhibitors” OR “dpp-4 inhibitor” OR dutogliptin OR alogliptin OR linagliptin OR saxagliptin OR sitagliptin OR vildagliptin) AND (“type 1 diabetes” OR t1dm). Publications were searched in Medline and Embase using the same strategy on one website (http://www.embase.com/). Additionally, all references of the articles downloaded from these databases were rapidly checked, and we manually searched 360 relevant articles. We did not employ any restrictions on publication year, specimen, or language.

### 2.3. Eligibility Criteria

We used the following selection criteria to ensure the inclusion of all relevant studies: (1) controlled clinical studies on the topic of DPP-4 inhibitors; (2) studies in which subjects were restricted to a diagnosis of type 1 diabetes, including patients with latent autoimmune diabetes in adults (LADA); and (3) studies reporting changes in control and experimental groups after treatment with respect to at least one indicator (HbA1c, insulin dosage, or blood glucose).

The exclusion criteria were as follows: (1) studies that did not meet any of the above inclusion criteria; (2) comments, reviews, abstracts, and articles other than original papers; and (3) duplicated data from the same laboratory.

Two investigators (QX Wang and M Long) independently searched the titles and abstracts as well as the full text, if necessary. All ambiguities were clarified by discussion with a third investigator (HT Zheng).

### 2.4. Data Extraction

The outcomes were recorded in detail. The primary outcome parameters studied here were classified as variations in *β*-cell function (detectable C-peptide levels) and glycemic control (the HbA1c, fasting blood glucose, and 2-hour postprandial blood glucose levels, as well as the area under the curve (AUC) for the blood glucose levels during a specific period). Insulin dosage, cholesterol, triglycerides, and adverse events were classified as secondary outcomes. We extracted the following data: treatment option, length of study, sample size, average age, duration of T1DM, body mass index (BMI), study design, and number of research centers. Briefly, all relevant data available that might impact the results were extracted and input into a predesigned table.

### 2.5. Quality Assessment

We used the Cochrane Risk of Bias Tool for Randomized Controlled Trials to assess the quality of the literature and the risk of bias.

### 2.6. Data Synthesis

We used the change in the value of the outcome to evaluate the therapeutic effect and emphasize the benefits of the combination of DPP-4 inhibitors with insulin versus insulin monotherapy. Because some articles reported the sample sizes (*N*), means (*M*), and standard deviations (SD) for females and males, we adopted the formula from chapter 7.7.3 of the Cochrane Handbook to synthesize *M*_1+2_ and SD_1+2_ data for systematic review. The formulas were

(1) $$\begin{array}{c} M_{1+2}=\frac{N_{1}M_{1}+N_{2}M_{2}}{N_{1}+N_{2}},\\ {SD}_{1+2}=\sqrt{\frac{\left(N_{1}-1\right){{SD}_{1}}^{2}+\left(N_{2}-1\right){{SD}_{2}}^{2}+\left(N_{1}N_{2}/\left(N_{1}+N_{2}\right)\right)\left({M_{1}}^{2}+{M_{2}}^{2}-2M_{1}M_{2}\right)}{N_{1}+N_{2}-1}}. \end{array}$$

We adopted the following formula to assess continuous variation in parameters, such as the HbA1c levels:

(2) $$\begin{array}{c} {SD}_{E,change}=\sqrt{{{SD}_{E,baseline}}^{2}+S{D_{E,final}}^{2}-2\times Corr\times {{SD}_{E,baseline}}^{2}\times {{SD}_{E,final}}^{2}}. \end{array}$$

### 2.7. Data Analysis

The analysis software Manager Review Version 5.2 (Cochrane Collaboration, Software Update, Oxford, UK) was used to synthesize the data, and 95% confidence intervals (95% CI) were calculated. We reported the mean difference (MD) for indicators of efficacy, including the HbA1c, fasting blood glucose, and 2-hour postprandial blood glucose levels and other continuous variables. The relative risk ratio (RR) was used for dichotomous variables, such as indicators evaluating safety. If the indicators were reported in fewer than 3 studies, we summarized the result of each study individually. Statistical significance was set to an *α* of 0.05. Heterogeneity between trials was estimated using the *I*^2^ statistic [17]. If significant heterogeneity was observed between studies, the results were reported using a random-effects model, and a sensitivity analysis was performed using the “leave one out” approach to explore the source of the heterogeneity. Then, a subgroup analysis was conducted, if applicable.

## 3. Results

### 3.1. Literature Retrieval

**Figure 1:**
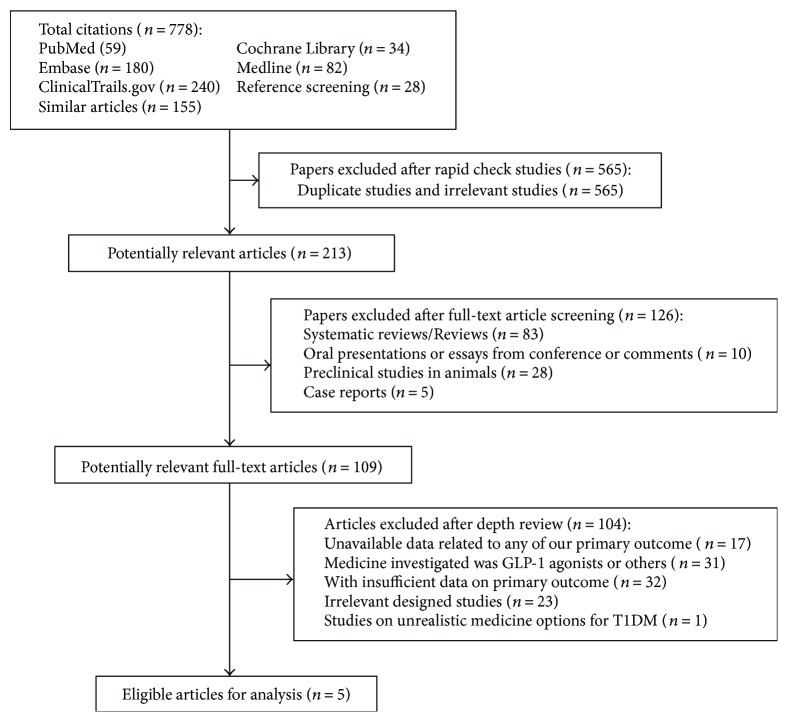
Flowchart illustrating the article screening process.

The literature retrieval process is shown in Figure 1. Two investigators searched the databases, and all relevant lists of studies were retrieved. Together with the reference lists from similar studies, a total of 778 titles and their keywords were identified. After a rapid check, we excluded 565 articles that were either duplicated or obviously irrelevant. Then, we screened the potential relevant full texts among the remaining 213 articles. By excluding inappropriate types of articles or studies researching undesired populations after an in-depth screen of the full texts, we obtained 109 potential articles. After we repeatedly studied these chosen articles and discussed their inclusion with Hongting Zheng, 5 randomized controlled trials (RCTs) were eventually considered eligible for this meta-analysis and systematic review.

Two hundred fifty-three participants, including 120 C-peptide-positive patients, from 5 RCTs were included in this meta-analysis and systematic review. Regarding medicine options, four studies [8, 18–20] examined the effects of combination therapy comprising sitagliptin and insulin compared with insulin monotherapy on T1DM, and the other study [13] investigated vildagliptin. Insulin injections were described in detail for both the experimental and control groups. Twelve patients from the study by Hari et al. [19] used an insulin analogue; conversely, patients from the other four studies used human insulin or an analogue. Only a few patients from the study by Garg et al. [20] received a continuous subcutaneous insulin infusion (CSII). Among the included studies, one study [18] was conducted on LADA patients. This study was designed to determine whether sitagliptin protected islet *β*-cells in patients with LADA. Regarding indicators, the earliest initiated study was an 8-week crossover study from Sweden [13] that focused on the modulation of glucagon levels by vildagliptin. Two studies [18, 19] focused on changes in the levels of hormones regulating glycemia after DPP-4 inhibitor treatment of patients with new-onset T1DM to explore the advantages of a sitagliptin and insulin combination compared with insulin monotherapy. A multicenter, randomized, placebo-controlled clinical trial from the United States [8] aimed to test the efficacy of the combination of lansoprazole with sitagliptin on T1DM. A randomized, double-blind, placebo-controlled trial with the largest sample size (125 subjects) conducted in the United States [20] aimed to explore the effect of sitagliptin on the postprandial glucagon and GLP-1 levels.  Table 1 shows the characteristics of the included studies. We also examined the inclusion and exclusion criteria for the participants enrolled in each study.

### 3.2. Assessment of Study Quality

**Figure 2:**
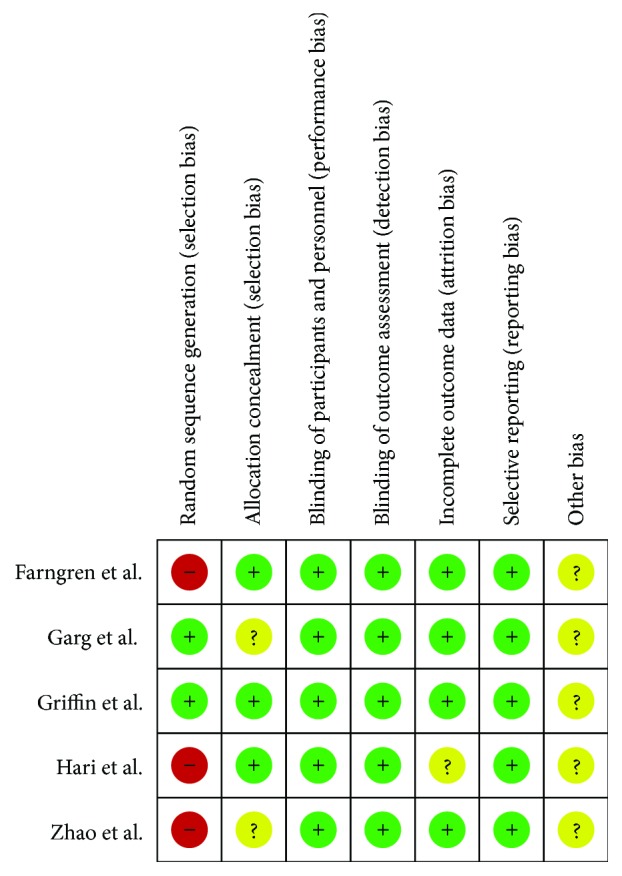
Risk of bias summary of included studies.

We evaluated the risk of bias using Review Manager version 5.2.  Figure 2 illustrates the summarized risk of bias. Three studies had a risk of selection bias attributed to random sequence generation. None of the studies had a high risk of selection bias (allocation concealment), reporting bias, performance bias, detection bias, or attrition bias. The results were unclear regarding whether other biases were present.

### 3.3. Results of the Meta-Analysis and Systematic Review

#### 3.3.1. Primary Outcomes

*(1) β-Cell Function: C-Peptide*. The additional effects of DPP-4 inhibitors on the C-peptide level remain controversial. One study [18] reported a positive effect and concluded that sitagliptin at least attenuated the progressive decrease in the C-peptide levels. Three studies [8, 19, 20] did not observe a notable effect, and one study [13] did not address this subject. The study by Zhao et al. [18] selected patients with recent-onset LADA who presented with a fasting C-peptide level (FCP) ≥ 200 or a 2-hour postprandial C-peptide (2 h CP) level ≥ 400 pmol/L. After 1 year of treatment, the participants who were administered sitagliptin and insulin had no evident decrease in their fasting C-peptide levels, 2-hour postprandial C-peptide levels, or ΔCP (ΔCP = 2hCP−FCP), whereas the patients administered insulin monotherapy displayed significantly decreased FCP, 2hCP, and ΔCP values. As shown in the study by Hari et al. [19], among patients with new-onset T1DM who presented with stimulated C-peptide levels ≥ 0.1 ng/mL, C-peptide was elevated by 0.0067 ± 0.19 ng/mL or −0.05 ± 0.28 ng/mL in the groups treated with sitagliptin alone or the combination of sitagliptin and insulin, respectively. However, neither the changes in the C-peptide levels in the groups nor between the groups were significant (*P* > 0.05). In the study by Griffin et al. [8], the AUCs for the 2 hour C-peptide levels at 6 and 12 months declined significantly in the groups that received one year of combination therapy with sitagliptin and lansoprazole or the placebo control, but the difference between the treatment groups was insignificant. In contrast, only 8 (13%) patients in the experimental group and 12 (19%) patients in the control group had detectable C-peptide levels (above 16 pmol/L) among the participants enrolled in the study by Garg et al. [20], and the C-peptide-positive patients who were randomly allocated to receive sitagliptin had a nonsignificant trend toward decreased HbA1c levels, mean glucose levels, and duration of hyperglycemia. The data were not available for the C-peptide-negative patients. All 28 participants with T1DM enrolled in the study by Farngren et al. [13] were initially C-peptide-negative and antibody-positive, but the researchers did not evaluate whether the C-peptide levels increased. In conclusion, 120 patients with T1DM included in this systematic review were C-peptide-positive, and the increase in the fasting C-peptide level, which was measured as an indicator of *β*-cell function, could not be confirmed in the group treated with DPP-4 inhibitors. Therefore, whether patients with a higher baseline C-peptide level, a shorter duration, and differential insulin usage will benefit more from combination therapy with any DPP-4 inhibitor remains unknown.

**Figure 3:**
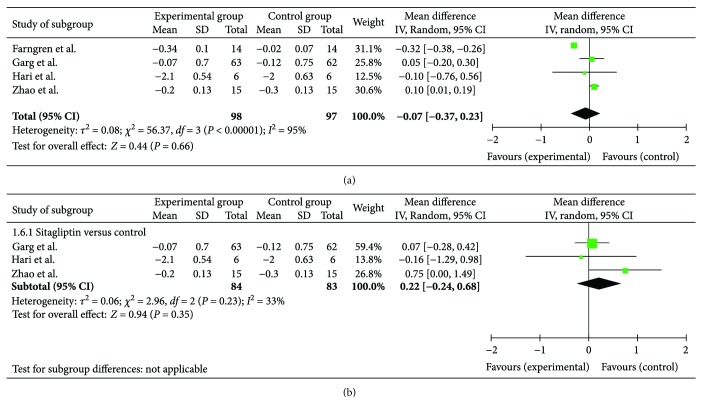
Forest plot of HbA1c.

*(2) Glycemic Control: HbA1c*. As a main outcome indicator, specific data on the HbA1c levels were available in four RCTs. Individually, three RCTs did not observe a significant improvement in the HbA1c levels when the patients were treated with sitagliptin [19, 20] or vildagliptin [13] in addition to insulin therapy. The authors in the other RCT [18] did not estimate the difference themselves, although the forest plot (shown in Figure 3(a)) showed an advantage for the HbA1c levels in the group that received combination treatment including sitagliptin over insulin monotherapy. Overall, a meta-analysis of these four studies (shown in Figure 3(a)) suggested that the addition of DPP-4 inhibitor treatment resulted in a greater decrease (0.07%) in the HbA1c level in patients with T1DM (95% CI: −0.37%–0.23%), albeit with significant heterogeneity. A sensitivity analysis was conducted to explore the sources of heterogeneity, and the heterogeneity became acceptable after the study by Farngren et al. [13] was removed. Thus, we hypothesize that the heterogeneity may be caused by different clinical characteristics, such as the study design (a single-center crossover study), negative baseline C-peptide status, and the use of different medicines (vildagliptin was used in Farngren et al.'s study, while the other studies used sitagliptin). Then, we performed a subgroup analysis. The vildagliptin subgroup showed advantages over insulin monotherapy in reducing HbA1c levels (0.32% (95% CI: −0.38%–0.26%)) in the study by Farngren et al. [13]. However, the subgroup of the other three studies on sitagliptin suggested that it was no better than insulin monotherapy. As shown in Figure 3(b), the change in the HbA1c levels in the experimental group was 0.22% (95% CI: −0.24%–0.68%) higher than the change in the control group, but the difference was not significant (*P* = 0.23, >0.05).

*(3) Glycemic Control: Glycemic Fluctuation*. Amazingly, after 16 weeks of treatment, the group receiving DPP-4 inhibitors had lower postprandial blood glucose levels and lower glucagon levels in the study by Garg et al., although the 4-hour AUCs for glycemia were not different between groups [20]. However, other studies did not provide evidence confirming the ability of DPP-4 inhibitors to improve the blood glucose levels. As shown in the study by Griffin et al., the AUCs for the 2-hour blood glucose levels increased by 2.04 mmol/L (95% CI: 1.09–2.99) in the group receiving sitagliptin and lansoprazole combined with insulin and by 3.05 mmol/L (95% CI: 1.17–4.92) in the insulin monotherapy group, but the difference between the groups was not significant [8]. Zhao et al. did not observe a significant difference in the fasting blood glucose or postprandial blood glucose levels between the two groups [18]. The remaining two studies [13, 19] did not report data on glycemic control.

#### 3.3.2. Secondary Outcomes

*(1) Other Relevant Indicators*. Regarding the insulin dosage, two studies [19, 20] provided raw data. The study by Hari et al. [19] revealed a significantly greater decrease in daily insulin consumption in the sitagliptin group (23.7 ± 13.9 U) than in the control group (15.2 ± 9.5 U) at the end of one year. According to the results from the other study [20], the changes in the total daily insulin dosage were −2.04 ± 11.45 U and 0.22 ± 9.17 U; however, the authors concluded that the change in the daily insulin dose (total units, units per kg/day, and basal and bolus units) did not change. Zhao et al. [18] reported a lack of difference in the insulin dosage at baseline and after treatment between groups. Farngren et al. [13] showed that the change in the insulin dosage was not significantly altered after treatment. Conversely, in the study by Griffin et al. [8], the insulin dosage increased over time in both groups and was significantly increased for the experimental group over time.

According to Garg et al. [20], the GLP-1 levels measured after 30 and 60 minutes in the sitagliptin group were significantly elevated after 16 weeks of treatment, whereas the GIP level measured at the 1-hour time point in the meal challenge test decreased. No differences in other relevant indicators, including the BMI, body weight, and lipid profiles, were observed between the experimental groups that received an additional DPP-4 inhibitor treatment and the control groups. Zhao et al. [18] did not observe significant differences in the BMI, weight, or lipid profiles (including low-density lipoprotein cholesterol, high-density lipoprotein cholesterol, triglycerides, and total cholesterol) between the treatment groups after treatment with sitagliptin for 12 months. In the study by Hari et al. [19], the BMI was significantly increased (1.5 ± 2.15 kg/m^2^, *P* < 0.05) in the insulin monotherapy group, and the body weight was also increased but did not significantly differ between groups. Garg et al. [20] consistently reported a lack of significant differences in the body weight or BMI between groups.

*(2) Overall Adverse Events and Side Effects*. We believe that hypoglycemia is one vital adverse event that deserves more attention when antidiabetic agents are added to insulin therapy. The study by Hari et al. [19] observed a high incidence of hypoglycemia, including severe hypoglycemia (4/6 versus 3/6 in the sitagliptin group compared with the insulin monotherapy group). Griffin et al. [8] reported adverse events of severe hypoglycemia (<2.06 mmol/L) in 11 (24%) and 7 (32%) of the patients in the experimental and control groups, respectively. Additionally, hypoglycemia (<3.33 mmol/L) occurred in 44 (96%) and 19 (86%) patients in these two groups, respectively. Only one patient in the control group experienced severe hypoglycemia and required an insulin dosage adjustment after a period of 16 weeks in Garg et al.'s study [20]. In the study by Zhao et al. [18], a low incidence of hypoglycemia (<3.9 mmol/L) occurred, and no severe hypoglycemia was reported. Finally, none of these studies observed a significant difference in the incidence of hypoglycemia. We tried to obtain information regarding the time spent in hypoglycemia, but the studies included in the present meta-analysis did not report this information. In another study [13], hypoglycemia was designed to be induced by a hyperinsulinemic hypoglycemic clamp and therefore was not valued as an adverse event. These authors revealed that the most common adverse event was a common cold, and no serious adverse events occurred. Because DPP-4 inhibitors elevate the endogenous GLP-1 levels, we also examined the incidence of pancreatitis. Three studies [8, 19, 20] emphasized the exclusion of patients with a history of pancreatitis or an increased risk of pancreatitis. Importantly, no pancreatitis events were reported throughout the periods of any of the included studies. Overall side effects that seemed to occur at a greater frequency in the experimental group included gastrointestinal disorders; infections and infestations; injury; poisoning; procedural complications; nervous system disorders; respiratory, thoracic, and mediastinal disorders; and skin and subcutaneous tissue disorders, but the frequencies were not statistically significant [8]. Records of adverse effects from Garg et al. [20] included a skin rash and a need for esophageal cancer surgery that finally led to treatment interruption. For most of the studies included in the present review, significant differences in the incidences of various adverse events were not observed between the two groups. All authors concluded that no serious side effects were confirmed to be clearly related to the DPP-4 inhibitors, including hypoglycemia, and other causes led to discontinuation from further participation; in addition, ketoacidosis and pancreatitis were not reported.

## 4. Discussion

Due to their recommended use as first-line antidiabetic agents by the American Association of Clinical Endocrinologists (AACE), DPP-4 inhibitors are currently widely used as outstanding monotherapeutic or combination therapeutic agents for T2DM. However, no DPP-4 inhibitor for T1DM has received authorization for application. No currently available treatment has shown lasting disease remission, although insulin is life-saving. Meanwhile, the life expectancy of patients with T1DM has substantially improved in recent decades. Therefore, additional management strategies are needed. Any novel treatment option (newer) in addition to insulin may produce a qualitative leap. Emerging studies have reported evidence that an elevation of serum DPP-4 activity is related to the pancreatic autoimmune process, intestinal proinflammatory alterations, and hepatobiliary injury [21–23] (i.e., DPP-4 may contribute to T1DM pathology). Additionally, inhibition of DPP-4 activity has shown a potential therapeutic effect in patients with T1DM. In fundamental studies, inhibition of DPP-4 activity alleviated insulitis and stimulated *β*-cell proliferation [10], markedly increased the *β*-cell mass and number of proliferating *β*-cells [24], elevated the glucose-stimulated insulin and C-peptide concentrations [25], and increased the total insulin levels [26], eventually delaying the diabetogenic autoimmune response [27, 28]. DPP-4 inhibitors (P32/98, DA1229, and MK0431) substantially decreased the blood glucose levels in animal models of T1DM [29–31] and even resulted in normoglycemia in some cases [32]. Moreover, DPP-4 inhibition increased the survival of islet grafts [33] and improved diabetic retinopathy [34]. According to clinical studies, patients with T1DM displayed higher DPP-4 levels [35, 36]. Consequently, we believe that treatment with DPP-4 inhibitors is a practical therapeutic strategy for T1DM, and thus, we sought to identify evidence from clinical practice. Numerous studies have provided evidence for the therapeutic effect of DPP-4 inhibitors in patients with T1DM. In clinical studies, DPP-4 inhibitors reduced the prandial insulin dose and its daily dosage [13, 37], inhibited glucagon secretion [13, 15], and decreased the blood glucose levels in patients with T1DM (2-hour postprandial and 24-hour AUCs) [37]. However, some clinical studies did not show any obvious improvement in the blood glucose levels, AUCs of the C-peptide levels, or HbA1c levels [18, 20]. Therefore, the therapeutic effects of DPP-4 inhibitors on T1DM remain controversial, and a comprehensive conclusion must be drawn after summarizing the currently available evidence.

Here, we systematically reviewed all relevant publications and performed a meta-analysis of five randomized controlled clinical trials on the effects of DPP-4 inhibitors on T1DM. We were also interested in another clinical trial (NCT00622284), which was originally intended to investigate the efficacy of linagliptin compared to glimepiride administration for 104 weeks as an add-on therapy to preferably >1500 mg of metformin in patients with T2DM with insufficient glycemic control. Some of the participants were reclassified as LADA depending on the tested autoantibodies during the trial. Further investigation found that these LADA patients demonstrated progressively increasing C-peptide levels and slightly decreasing HbA1c levels after linagliptin-metformin treatment compared to the baseline levels [38], hinting at a possible therapeutic effect of DPP-4 inhibitors on T1DM. Considering the heterogeneity, we excluded this study from our meta-analysis. Based on the pooled data in our meta-analysis, treatment with DPP-4 inhibitors neither resulted in a significantly greater decrease in the HbA1c levels nor further reduced the insulin dosage or affected the weight or BMI in patients with T1DM. Unfortunately, we were unable to perform a pooled analysis of the data on the fasting blood glucose or prandial blood glucose levels because the raw data or changes were not available for some studies. Although benefits, including declining fasting blood glucose levels [39], prandial blood glucose levels [13, 20, 37], AUCs of 24-hour blood glucose levels [37], and HbA1c levels, as well as reductions in the insulin dosages [37], have been reported by individual studies, these effects failed to be revealed in other similar studies. The discrepancy may be caused by different baseline characteristics (such as C-peptide levels) of the enrolled populations, different lengths of follow up [39], and other variables. DPP-4 inhibitors significantly increased the GLP-1 and GIP levels [13, 14, 40], reduced the glucagon levels during hyperglycemia, and sustained glucagon counterregulation during hypoglycemia in patients with T1DM [13, 14]. Fortunately, the absence of relevant severe side effects promises more studies regarding security to validate the therapeutic effects.

The limitations of this review include the small number of RCTs and the absence of some data. More desirable studies and raw data will enable us to perform a stratified analysis and further explore the roles of DPP-4 inhibitors as treatments for T1DM. Another limitation is that immunological indicators have not been reported in any studies, although conclusive evidence has been established for the immunoregulatory effects of DPP-4 inhibitors. In addition, almost none of the studies included in this review reported the full-scale adverse effects that were reported during the treatment of T2DM, such as cardiovascular events and tumors, which were the most common, severe, and vital adverse effects [41]. Pharmacotherapy for T1DM is required throughout the patient's lifetime; therefore, the application of any novel medicine requires a very thorough long-term cost/benefit analysis (i.e., for cancer and other relevant endpoints). Therefore, we believe that a lack of a cost/benefit analysis is one limitation.

As discussed above, more studies should be recruited to elucidate the roles of DPP-4 inhibitors in appropriately selected patients with T1DM. A larger sample size, a longer follow-up, unified characteristics of participants (such as diabetes duration and C-peptide levels), the comprehensive monitoring of glycemia and relevant hormone levels, the use of immunological indicators (such as CD4^+^ and CD8^+^ T cells), and a report of full-scale adverse effects should be the focus of future studies.

## Figures and Tables

**Table 1:** Characteristics of the included studies.

Studies	Hari et al. (NCT01235819)	Griffin et al. (NCT01155284)	Garg et al. (NCT01227460)	Zhao et al. (NCT01159847)	Farngren et al. (NCT01147276)
Experimental treatment	Sitagliptin (100 mg, qd) + insulin^a^ (premix insulin)	Sitagliptin (100/50 mg, qd) + lansoprazole (60/30 mg, qd) + insulin (unspecified)	Sitagliptin (100 mg, qd) + insulin^b^ (premixed insulin excluded)	Sitagliptin (100 mg, qd) + insulin (unspecified)	Vildagliptin (50 mg, bid) + insulin^c^ (long-acting and short-acting insulin)
Control treatment^d^	Insulin (bid-tid)	Placebo + insulin	Placebo + insulin	Insulin	Placebo + insulin
Treatment period (weeks)	52	52	16	52	8
Experimental samples	6	40	63	15	14
Control samples	6	18	62	15	14
Average age (years)	27.15 ± 4.20	16.18 ± 5.78	37.99 ± 14.01	47.45 ± 3.27	U
Mean duration of T1DM	33.25 ± 9.37 d	103.41 ± 51.74 d	21.01 ± 11.00 y	1.4 ± 0.20 y	11.0 ± 4.30 y
Average BMI (kg/m^2^)	21.50 ± 3.39	21.51 ± 3.95	27.45 ± 4.54	23.35 ± 0.85	24.8 ± 3.30
Design	RCT	RCT	RCT	RCT	RCT
Number of centers	1	3	3	1	1
HbA1c at baseline (%)	9.75 ± 0.83	7.18 ± 1.09	8.55 ± 0.70	6.45 ± 0.20	7.49 ± 0.55
Cholesterol at baseline (mmol/L)	U	U	U	4.65 ± 0.16	U
C-peptide at baseline (pmol/L)	0.395 ± 0.15^e^	685.44 ± 412.36	≥16 pmol/L^f^	401.75 ± 52.27^g^	U
LDL-C at baseline (mmol/L)	U	U	U	2.75 ± 0.13	U
Triglyceride at baseline (mmol/L)	U	U	U	1.2 ± 0.1	U
Inclusion criteria	Recent T1DM < 3 m, GAD^+^or stimulated CP < 0.5 ng/mL, age < 30 y	Aged 11–36 y, <6 m	Aged 18–80 y, after diagnosed for 1 y, BMI < 35 kg/m^2^	Aged 25–70 y, FCP ≥ 200/2 hCP ≥ 400 pmol/L, duration < 3 y, LADA	Age > 18 y, duration of 2–20 y, HbA1c of 6.5–8.5%
Exclusion criteria	C-peptide < 0.1 ng/mL, age > 30 y, pancreatic disease	Risk of pancreatitis, pregnant	BMI ≥ 35 kg/m^2^	Other autoimmune diseases, insulin > 0.8 U/kg/d, renal disease	Pregnant or lactating, acute infection, liver disease, blood donor, using GH or oral steroid

d: days; m: months; y: years. Data are presented as the mean ± SD or as numbers (percentages). U: unknown; NG: not given; GH: growth hormone; BMI: body mass index; RCT: randomized controlled trial. ^a^Patients initially were started on a twice daily premixed insulin regime (25% insulin Lispro and 75% insulin Lispro protamine) and later shifted to a three-times-daily premixed regime depending on their glycemic profiles. ^b^Patients may be using insulin via a continuous subcutaneous insulin infusion (CSII) or multiple daily injections (MDI) containing bolus and basal insulin. ^c^Twenty-six patients were treated with daily basal-bolus injections; their mean insulin dose was 30 U/d (long-acting insulin; 0.37 ± 0.07 U/kg) and 31 U/d (short-acting insulin; 0.37 ± 0.09 U/kg). Two patients were treated with a continuous sc insulin infusion [daily dose 60 and 36 U (0.67 and 0.52 U/kg), resp.]. ^d^The insulin dosages used were consistent with the experimental group. ^e^ng/mL; ^f^only 20 patients were reported to be C-peptide-positive; ^g^fasting C-peptide level.
